# Peat deposits store more carbon than trees in forested peatlands of the boreal biome

**DOI:** 10.1038/s41598-021-82004-x

**Published:** 2021-01-29

**Authors:** Joannie Beaulne, Michelle Garneau, Gabriel Magnan, Étienne Boucher

**Affiliations:** 1 Geotop Research Center, Université du Québec à Montréal, Montréal, QC H3C 3P8 Canada; 2 Department of Geography, Université du Québec à Montréal, Montréal, QC H3C 3P8 Canada; 3 GRIL-UQAM, Université du Québec à Montréal, Montréal, QC H3C 3P8 Canada; 4 Centre d’études nordiques, Université Laval, Québec, QC G1V 0A6 Canada

**Keywords:** Carbon cycle, Boreal ecology, Palaeoecology, Wetlands ecology, Climate-change mitigation

## Abstract

Peatlands are significant carbon (C) stores, playing a key role in nature-based climate change mitigation. While the effectiveness of non-forested peatlands as C reservoirs is increasingly recognized, the C sequestration function of forested peatlands remains poorly documented, despite their widespread distribution. Here, we evaluate the C sequestration potential of pristine boreal forested peatlands over both recent and millennial timescales. C stock estimates reveal that most of the carbon stored in these ecosystems is found in organic horizons (22.6–66.0 kg m^−2^), whereas tree C mass (2.8–5.7 kg m^−2^) decreases with thickening peat. For the first time, we compare the boreal C storage capacities of peat layers and tree biomass on the same timescale, showing that organic horizons (11.0–12.6 kg m^−2^) can store more carbon than tree aboveground and belowground biomass (2.8–5.7 kg m^−2^) even over a short time period (last 200 years). We also show that forested peatlands have similar recent rates of C accumulation to boreal non-forested peatlands but lower long-term rates, suggesting higher decay and more important peat layer combustion during fire events. Our findings highlight the significance of forested peatlands for C sequestration and suggest that greater consideration should be given to peat C stores in national greenhouse gas inventories and conservation policies.

## Introduction

Terrestrial vegetation is a key component in global climate cycles through its capacity for carbon (C) sequestration^1^. In the boreal biome, forests cover ~ 8% of the land area and sequester approximately 272 ± 23 Gt of carbon^2^, while northern peatlands store an estimated 415 ± 150 Gt of carbon, covering only ~ 2% of the global land surface^3^.

In the Northern Hemisphere, peatland development has mostly been attributed to the paludification process, which led to the establishment of open or forested peatlands depending on drainage and the initial presence of forest vegetation^4,5^. Boreal forested peatlands are characterised by a closed to semi-open canopy cover and an organic layer thickness larger than 30 cm^6–8^. Recent methodological mapping improvements revealed that the coverage of forested peatlands has most likely been underestimated. In Canada, for example, preliminary estimates suggested that between 10 and 17% of northern peatlands were forested^8^, but new estimates indicate that these ecosystems probably cover up to 50% of the Canadian peatland area in the boreal and subarctic biomes^9,10^. While these ecosystems are mostly pristine sites in Canada, over 10 million ha of peatlands have been drained and managed for forestry in Fennoscandia and Russia^11^. Carbon sequestration in drained forested peatlands has been largely studied in Finland (e.g.,^12–15^), but the global C sequestration potential of pristine boreal forested peatlands remains poorly documented, despite their widespread distribution.

Estimation of C storage capacities in the boreal biome has recently been biased towards aboveground forest-related components, while belowground components remained neglected. For example, climate change mitigation efforts tend to focalize on evaluating the role and importance of forests as carbon sinks for anthropogenic C emissions sequestration (e.g.,^16–19^). From that point of view, boreal forested peatlands may have been overlooked as unproductive ecosystems, due to their semi-open structure and low stem density. Despite the recommendations of the *IPCC Special Report on Climate Change and Land*^20^ for reducing deforestation and increasing C storage by preserving peatland ecosystems, there remains a lack of consideration for organic layer C stores in national greenhouse gas inventories and conservation policies^21–23^. As a result, the C sequestration potential of forested peatlands is inaccurately evaluated and their role in climate mitigation is certainly underestimated.

Recently, Magnan et al.^24^ contributed to documenting C dynamics in boreal forested peatlands by investigating their long-term C storage capacities. Through a comparison of C stocks between tree aboveground biomass and peat layers, they showed that organic horizons—accumulated over millennia—store significantly more carbon than mature black spruce trees. However, contrasting timescales associated with peat accumulation and forest maturation make comparing these two components difficult in terms of their relative effectiveness as C stores. This highlights that comparing forests and peatland ecosystems in terms of C sequestration is a non-trivial task. There is an important need to document C storage capacities of trees and peatlands on common timescales to better inform decisions on ecosystem management and nature-based solutions for climate change mitigation.

**Figure 1:**
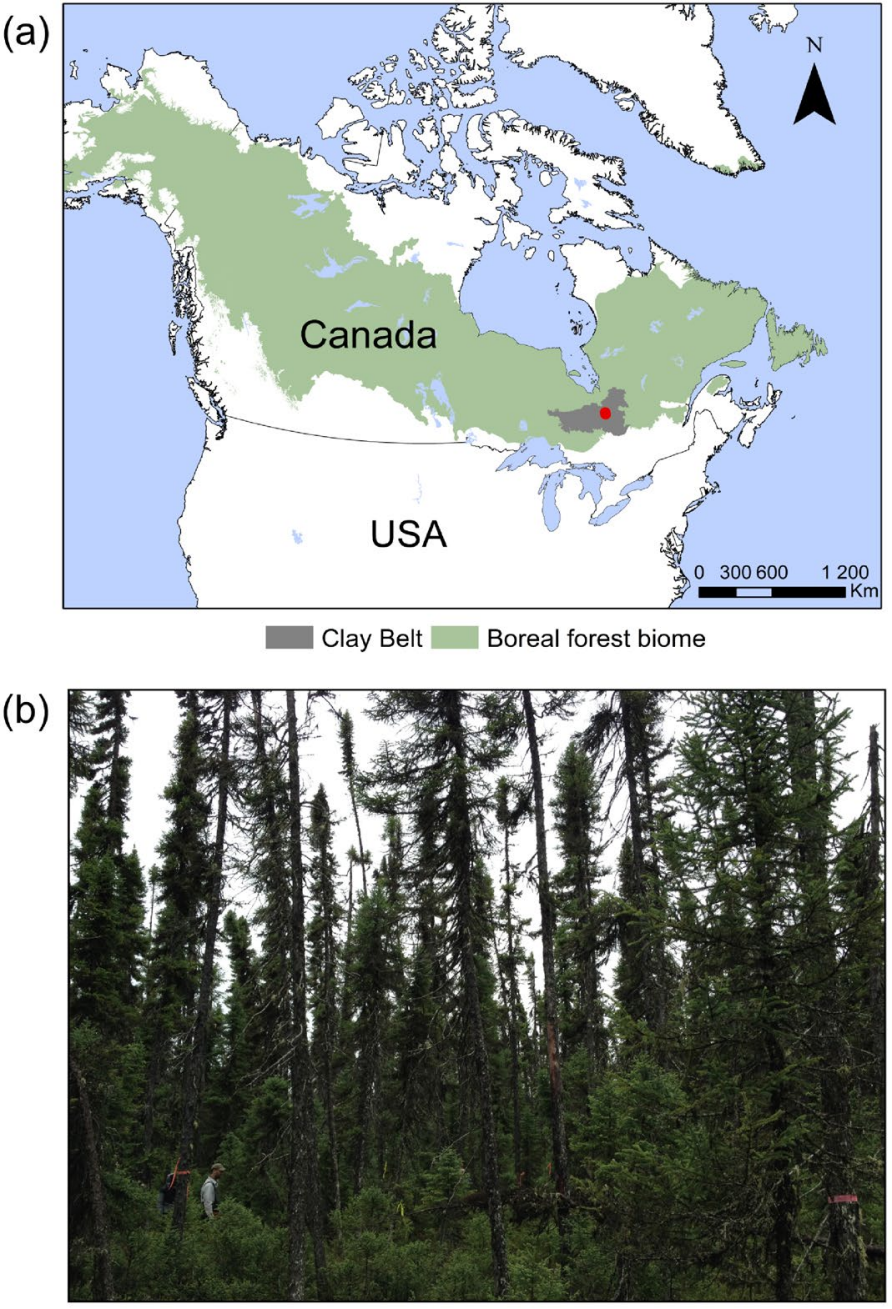
(**a**) Location of the studied boreal forested peatland (red dot) in the Clay Belt of eastern Canada (map generated using ArcGIS 10.7.1—https://desktop.arcgis.com/). (**b**) Photograph of the sampling site CAS100.

Here, we investigate C sequestration in pristine boreal forested peatlands over both recent and millennial timescales—which respectively correspond to the timeframe of tree growth (last two centuries) and that of peat accumulation—by combining paleoecological and dendrochronological approaches. To highlight the potential value of C sink capacities, we compared C stocks in tree biomass and peat layers by using a high temporal resolution approach in a forested peatland in eastern Canada (Fig. 1). To our knowledge, there has not been yet such a comparison on C stock quantification in the boreal biome.

## Results

### Peat and tree chronologies

**Figure 2:**
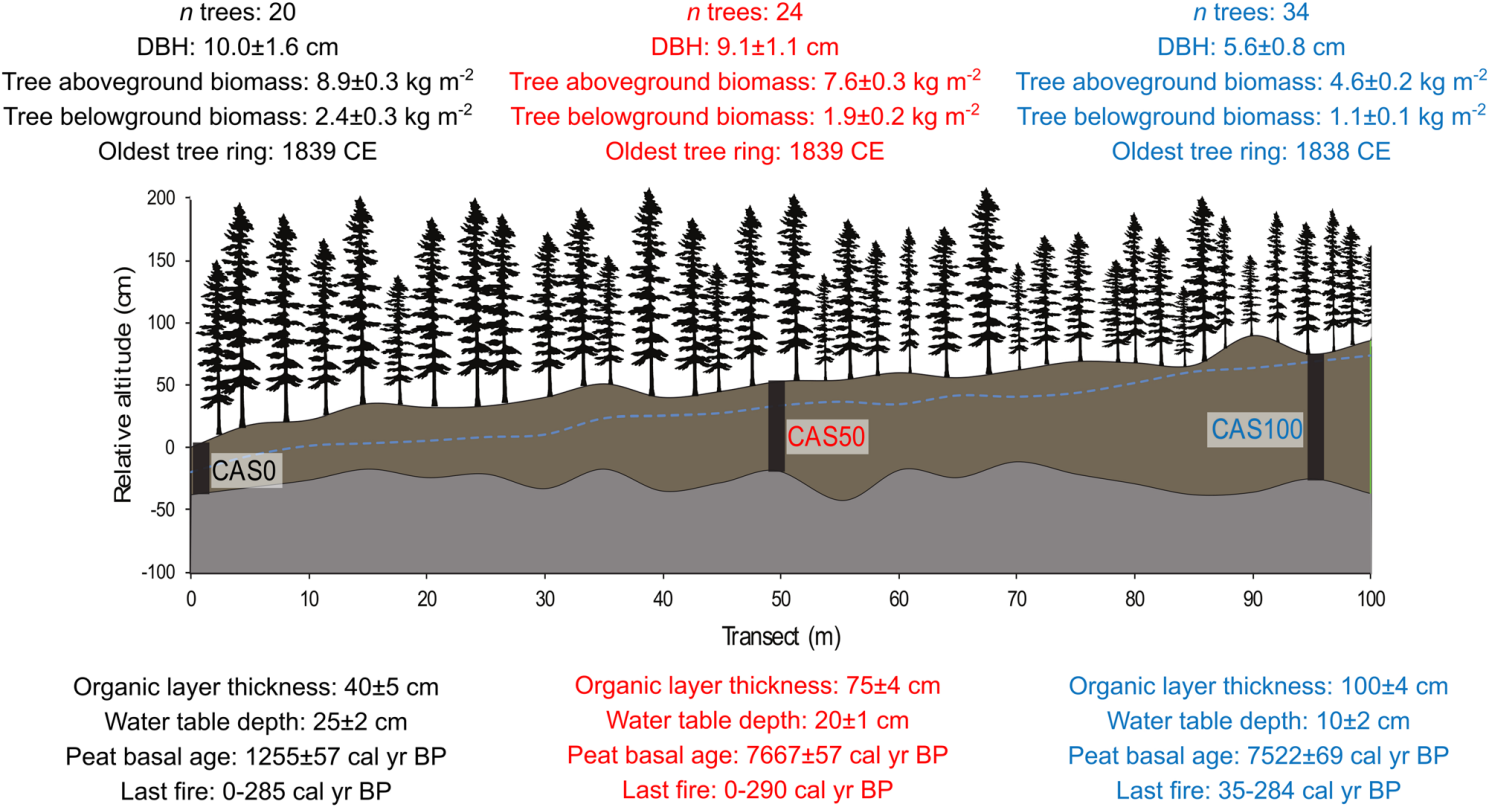
Schematic representation of the transect and characteristics of the three study sites. The relative altitude of the organic layer and the mineral surfaces are shown in brown and grey, respectively. Black rectangles correspond to the peat cores sampled and the dotted blue line illustrates the water table level measured on the field. Trees are not to scale but are representative of the variation in canopy openness with peat thickening. Tree counting was performed within a 10 × 10 m plot at each site. Standard errors are shown by ± values. Drawing was generated using Inkscape 0.92.

**Figure 3:**
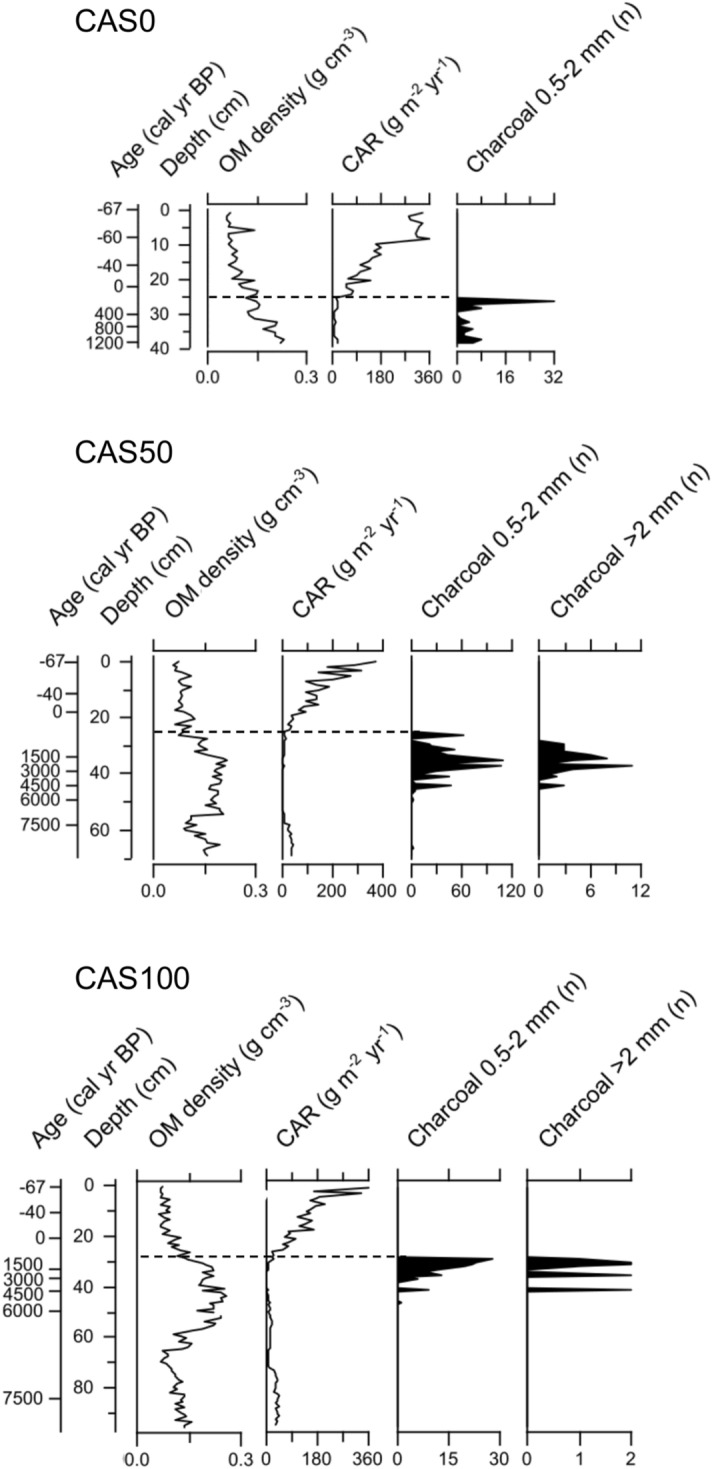
Peat organic matter (OM) density, carbon accumulation rates (CAR) and charcoal records^26^ from the three studied peat cores along the transect. Dashed lines indicate the last fire event above which recent apparent rates of C accumulation (RERCA) were calculated. The detailed plant macrofossil data for these cores are presented in^26^.

To document tree and peat C stocks in boreal forested peatlands, we established a transect with an increase in organic layer thickness reflecting different degrees of paludification. The selected study sites CAS0, CAS50 and CAS100 along the transect have a mean organic layer thickness of 40, 75 and 100 cm, respectively (Fig. 2). Radiocarbon (^14^C) dating revealed that peat initiation started around 1200 cal year BP at CAS0 and around 7600 cal year BP at both CAS50 and CAS100 (Table S1). Charcoal fragment counting suggests multiple fire events between 7000 and 150 cal year BP (Fig. 3). The ^14^C dating of the most recent charcoal layer of each site indicated that the median calibrated age of the last fire event ranged between 175 and 179 cal year BP. These ages were validated by ^210^Pb dating (Table S2). Age-depth models were developed for each site by combining ^14^C and ^210^Pb chronologies (Fig. S1).

The combination of peat dating and tree-ring chronologies confirms that the last fire occurred around 200–250 years ago (~ 1800 CE). Tree-ring analyses performed on twenty black spruce (*Picea mariana* (Mill.) BSP) trees per site revealed the presence of even-aged stands. Tree chronologies cover the period 1839–2018 CE at CAS0 and CAS50, and the period 1838–2018 CE at CAS100 (last 180 years). Considering that age might be underestimated for trees growing in thick organic substrate^25^, these results strongly suggest the establishment of black spruce immediately after the fire.

The vegetation succession of the studied sites is described in Beaulne et al.^26^. Over the last two centuries, the canopy opening, which resulted from the last fire, first allowed the establishment and the expansion of *Sphagnum* mosses at the three sites. The black spruce post-fire regeneration then followed, leading to the present day black-spruce-*Sphagnum* dominated ecosystem.

### Tree biomass

**Figure 4:**
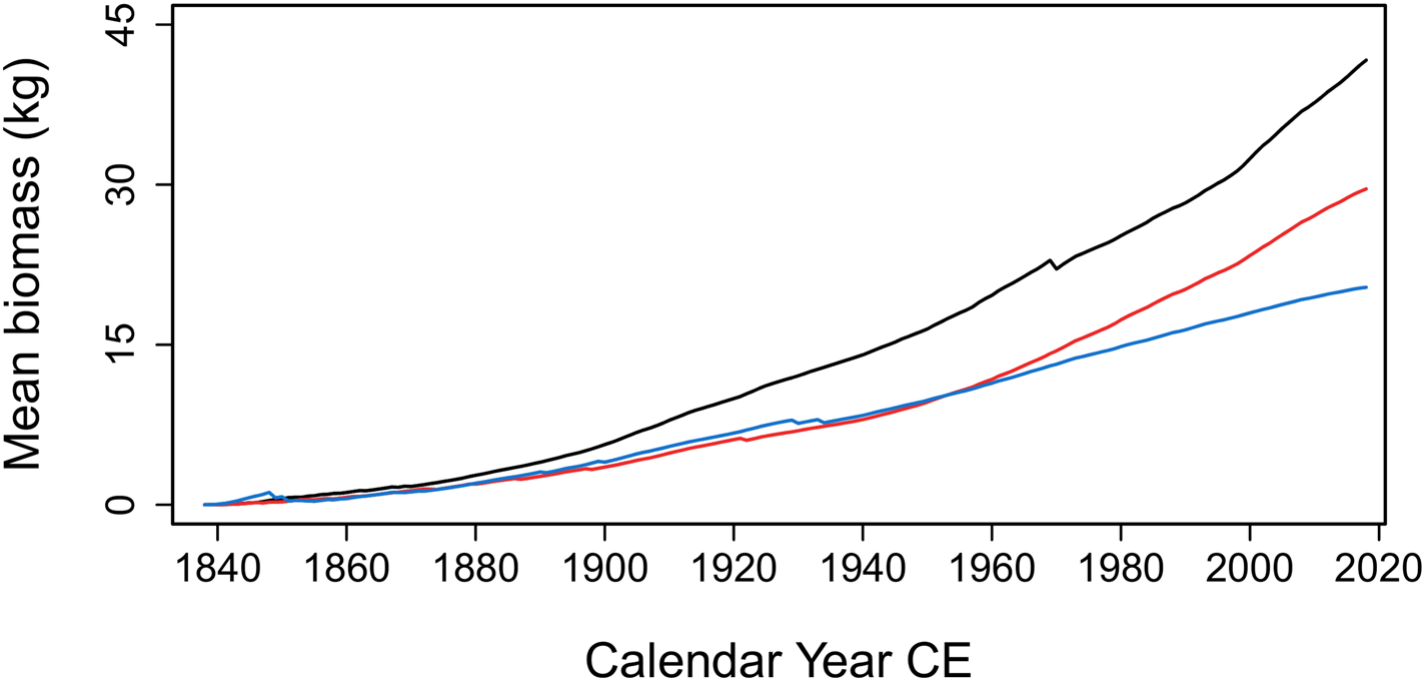
Mean annual cumulative aboveground biomass of twenty black spruce trees per site. Results from CAS0, CAS50 and CAS100 are presented in black, red, and blue, respectively.

Tree growth rates vary according to the degree of paludification. The diameter at breast height (DBH) values decrease along the transect with the organic layer thickening. Trees from sites CAS0, CAS50 and CAS100 have a mean DBH of 10.0, 9.1 and 5.6 cm, respectively (Fig. 2). Tree aboveground biomass reaches 8.9 kg m^−2^ at CAS0 and 7.6 kg m^−2^ at CAS50, but only 4.6 kg m^−2^ at CAS100. The mean annual cumulative aboveground biomass calculated from ring-width measurements of the twenty black spruce trees per site also indicates a reduction in tree growth rates along the paludification gradient (Fig. 4). CAS0 and CAS50 sites show similar exponential trends in biomass production, although growth was slower at CAS50. At CAS100, tree aboveground biomass progressed at a very similar rate to CAS50 until the 1960s. Thereafter, biomass production slowed down, increasing linearly rather than exponentially. At the end of the studied period (2018 CE), the mean tree aboveground biomass at CAS0 was two times higher than that of CAS100. Tree belowground biomass is estimated at 2.4 (2.0–3.0 kg m^−2^), 1.9 (1.6–2.3 kg m^−2^) and 1.1 kg m^−2^ (1.0–1.2 kg m^−2^) for CAS0, CAS50, and CAS100, respectively (Fig. 2).

### Carbon accumulation data

The highest long-term apparent rate of C accumulation (LORCA) is observed at CAS0 (18.0 g C m^−2^ year^−1^) where the youngest peat core was collected (Table 1). LORCA values are similar at CAS50 and CAS100, where peat initiation occurred around 7600 cal year BP, with a value of 6.0 and 8.8 g C m^−2^ year^−1^, respectively. Recent rates of C accumulation (RERCA) were calculated for the periods 1900 CE-present and 1950 CE-present, and correspond to the partly decomposed peat layers of the acrotelm. Post-1900 CE RERCA values are similar between the three sites, particularly at CAS0 and CAS50 (83.5 and 84.4 g C m^−2^ year^−1^; Table 1). RERCA values from 1950 CE to present are comparable at CAS0 and CAS100 (130.8 and 129.3 g C m^−2^ year^−1^ respectively), and slightly lower at CAS50 (117.1 g C m^−2^ year^−1^). The variability in apparent C accumulation rates (CAR) over time are similar between the three sites (Fig. 3). CAR values are higher than 100 g C m^−2^ year^−1^ in the first 20 cm of the peat cores dominated by *Sphagnum* spp., and decrease (< 5 g C m^−2^ year^−1^) between 30 and 50 cm at CAS50 and CAS100 where ligneous peat is dominant and highly decomposed.

**Table 1:** Long-term apparent rate of C accumulation (LORCA) and recent apparent rate of C accumulation (RERCA) data for the three study sites.

	CAS0	CAS50	CAS100
Core length (cm)	38	69	95
Basal age (cal year BP)	1255 ± 57	7667 ± 57	7522 ± 69
LORCA (g C m^−2^ year^−1^)	18.0 ± 0.8	6.0 ± 0.1	8.8 ± 0.1
RERCA 1900 CE (g C m^−2^ year^−1^)	83.5 ± 28.3	84.4 ± 6.3	91.3 ± 15.6
RERCA 1950 CE (g C m^−2^ year^−1^)	130.8 ± 7.6	117.1 ± 5.9	129.3 ± 6.5

**Figure 5:**
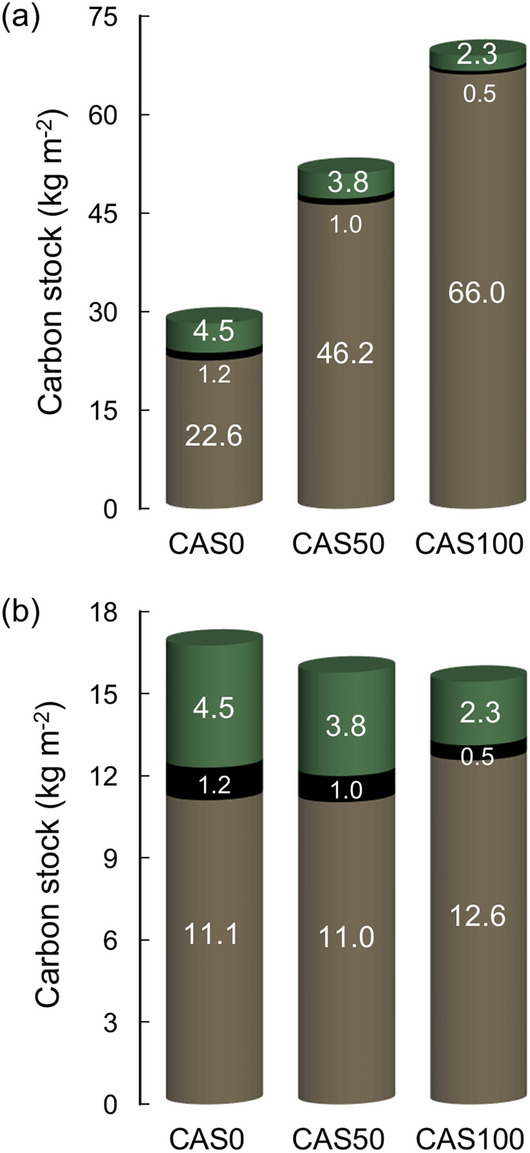
(**a**) C stocks accumulated since peat initiation and (**b**) the last 200 years (post-fire) in peat deposits (brown), tree belowground biomass (black), and tree aboveground biomass (green). Peat basal ages are 1255, 7667 and 7522 cal year BP for sites CAS0, CAS50 and CAS100, respectively. Tree minimal age is 180 years old for the three sites.

Tree C mass decreased with organic layer thickening. As a result, the most paludified site (CAS100) exhibited the highest peat C stock and the lowest tree C stock (Fig. 5a). Carbon stocks were significantly higher in peat (22.6–66.0 kg m^−2^) compared to tree aboveground and belowground biomass (2.8–5.7 kg m^−2^) for all sites—with peat layers currently containing between 4 to nearly 25 times more carbon than the tree components. Considering that organic layers have accumulated carbon during a much longer period than trees (Fig. 2), post-fire C stocks were calculated separately to obtain comparable values on the same temporal scale (i.e., ~ last 200 years). Therefore, tree C stocks were compared with peat C stocks above the last charcoal layer at each site. Again, C mass was much higher in these upper peat layers (first 25–27 cm), with a mean value of 11.6 kg m^−2^ compared to 4.4 kg m^−2^ in trees, resulting in C stocks up to almost five times higher in peat than in tree aboveground and belowground biomass (Fig. 5b).

## Discussion

This study confirms that most of the carbon stored in boreal forested peatlands is found in peat layers. The amount of carbon stored in peat deposits (22.6–66.0 kg m^−2^)—accumulated over millennial timescales—is comparatively much larger than the amount stored in aboveground and belowground tree biomass (2.8–5.7 kg m^−2^) (Fig. 5a). These results support the recent findings of Magnan et al*.*^24^ who also observed considerably higher C stocks in organic layers (62–172 kg m^−2^) than in tree aboveground biomass (1.5–5.3 kg m^−2^) in forested peatlands in eastern Canada. Our C stock comparison performed on an equivalent period of time (last ~ 200 years) revealed that organic layers (11.0–12.6 kg m^−2^) store more carbon than trees (2.8–5.7 kg m^−2^) even on a short timescale (Fig. 5b). While recent horizons will be subjected to further decomposition^27^—as will the tree biomass—this nevertheless suggests that belowground carbon storage is strikingly superior to its aboveground counterpart at all timescales. Moreover, in undisturbed forested peatlands, carbon from roots and deadwood biomass is likely to be eventually transferred in peat C pool^28^, although further knowledge is needed on long-term biomass transfer dynamics in these ecosystems.

Total C stocks (trees and peat) estimated in the study sites range from 28.3 to 68.8 kg m^−2^. Although the biomass of shrubs and deadwood was not included in our estimates, these data are higher than the C storage value of 23.9 kg m^−2^ calculated for boreal forests worldwide that includes all the ecosystem components^2^. These results support the statement that peatlands contain much more organic C than other terrestrial ecosystems^29^. Furthermore, the dominance and high water retention capacity of *Sphagnum* mosses in forested peatlands allows for the persistence of wet surface conditions, which prevent deep burning and thus limit carbon consumption during fire events^30,31^. *Sphagnum* mosses are also more recalcitrant to decomposition than vascular species^32,33^.

Long-term rates of C accumulation (LORCA) at the three study sites range from 6.0 to 18.0 g C m^−2^ year^−1^ (Table 1). These results are similar to those obtained by Magnan et al.^24^, ranging between 9.3 and 22.8 g C m^−2^ year^−1^. The highest LORCA, observed at CAS0, is consistent with the mean value of 17 g C m^−2^ year^−1^ reported by Zoltai and Martikainen^7^ for Canadian forested peatlands. However, LORCA values at CAS50 and CAS100 are more similar to the 8 g C m^−2^ year^−1^ calculated in black spruce forests of Alaska by Manies et al.^34^, and are among the lowest LORCA documented in boreal peatlands. The long-term C accumulation rates of all sites are lower than the global average of 22.9 g C m^−2^ year^−1^ from northern non-forested peatlands^35^ and the mean value of 26.1 g C m^−2^ year^−1^ documented in boreal and subarctic non-forested peatlands in eastern Canada^36^. Plant macrofossil analyses previously conducted on the three studied peat cores indicate the presence of dense and highly humified woody horizons^26^ (mean OM density: 0.14 g cm^−3^; Fig. 3) that suggest high decay rates compared to non-forested *Sphagnum* peatlands (mean OM density: 0.105 g cm^−3^)^35^—which likely explains the low rates of C sequestration over millennial timescales in these forested peatlands. This could be attributed to the persistently low water tables over millennia which favour higher aerobic decomposition rates in the upper peat layers—as observed by Magnan et al.^37^ from testate amoeba analysis in similar woody-dominated horizons in another forested peatland 15 km apart from the present study sites. The low LORCA values could also be attributed to important organic layer combustion during fire events, as forested peatlands have relatively high woody biomass as opposed to non-forested peatlands. Indeed, the lowest carbon accumulation rates (CAR) in the three peat cores correspond to ligneous horizons^26^ with particularly high organic matter density (> 0.15 g cm^−3^), in which numerous charcoal fragments were found (Fig. 3). Our results, supported by those from Magnan et al.^24^, suggest that boreal forested peatlands are less efficient C sinks over millennial timescales than non-forested peatlands.

Recent apparent rates of C accumulation (RERCA) range between 83.5 and 91.3 g C m^−2^ year^−1^ for 1900 CE-present and between 117.1 to 130.8 g C m^−2^ year^−1^ for 1950 CE-present (Table 1). While these recently accumulated peat layers will be subject to further decomposition, these RERCA values suggest high contemporaneous C uptake and storage by *Sphagnum* mosses in forested peatlands. These data are comparable with published RERCA in non-forested peatlands of eastern Canada. Turunen et al.^38^ calculated a mean RERCA of 126.5 g C m^−2^ year^−1^ for 23 peatlands (1953–2003) while Piilo et al.^39^ documented a RERCA of 85.94 and 102.50 g C m^−2^ year^−1^ in high boreal and subarctic peatlands for the periods 1900–2017 CE and 1950–2017 CE, respectively. These results show that boreal forested and non-forested peatlands can have similar carbon sequestration potential in a short-term perspective.

The ongoing paludification process at the study sites led to a decrease in tree aboveground biomass (Figs. 2–3). A decline in forest productivity with organic layer thickening has been commonly observed in the boreal biome (e.g.,^40–42^). Forested peatlands are widely managed by the forest industry to control, reduce or reverse the paludification process and increase forest site productivity through drainage, fertilization or mechanical site preparation^14,43–47^. These management practices—favoring organic layer decay—are performed with different considerations for the impact they generate on both atmospheric and aquatic CO_2_ and CH_4_ fluxes. The most extensive studies have been conducted in Finland^14,47,48^, while in Canada the C sequestration function of peat soils and carbon accounting are still rarely taken into account in forested peatland management^49,50^. Yet, the value of organic horizons for C sequestration highlighted in the present study suggests that the loss of carbon from peat layer disturbance or removal cannot be offset within a short-term period (e.g., 200 years) by enhanced tree growth. For example, at CAS0—which site shows relatively good tree growth rates—tree aboveground biomass would need to be five times larger to reach long-term organic layer C storage capacities (22.6 kg C m^−2^). Our results suggest that prioritising forest growth over peatland development and conservation can have important and permanent repercussions on the global carbon cycle on both short- and long-term perspectives.

Our study shows that the key C sequestration function of forested peatlands should be considered in forest management practices. Since it is not possible to optimize both forest productivity and soil carbon sequestration in these ecosystems, criteria based on site characteristics should be established to inform management decisions. Our results show that black spruce growth had similar exponential trends between the three sites up to the 1960s, where stem growth was thereafter more restrained at CAS100 (Fig. 4). Age-depth modelling indicated that the organic layer was 75 cm thick at CAS100 at that time. The two other sites, which showed relatively good growth trends, never reached that organic layer thickness. The slowdown in biomass production could be the result of the black spruce rooting zone fully migrating in the organic substrate^41^. While further studies are needed, this may be an indicator of the peat thickness threshold above which the protection of the C sequestration function of forested peatlands should be prioritised over tree productivity.

Through the first attempt to compare the relative efficiency of two different components for C storage over a common timescale in the boreal area, we show that peat can have higher C storage capacities than trees, even on a short-term perspective. Although additional studies including peat decomposition models may be needed, our study clearly demonstrates the effectiveness of peat soils for carbon sequestration over both recent and millennial timescales in boreal forested peatlands. Thus, our results highlight the important value of forested peatlands for carbon sequestration, but also the key role that these ecosystems can play in climate change mitigation strategies. Considering that tree restoration and afforestation practices may be expensive and that the fertilization effect of rising atmospheric CO_2_ concentration on tree growth is negligible (e.g.,^51–54^), greater consideration should be given to the conservation of peatland ecosystems, which naturally contribute to reaching climate mitigation goals.

## Methods

### Study area

Data were collected in eastern Canada within the Clay Belt region of the black spruce-feather moss bioclimatic domain, south of James Bay^55^ (Fig. 1a). This area is particularly prone to paludification due to the relatively cold and humid climate, the flat topography, and the dominance of poorly-drained clayey deposits left by the proglacial lakes Barlow and Ojibway^45,56^. Moreover, the regional fire cycle is estimated at ~ 400 years since 1920^57^, allowing the accumulation of thick organic layers between fire events. Mean annual temperature is 0.3 °C (1950–2013 period)—ranging from -18.9 °C in January to 16.3 °C in July—and mean annual precipitation is 818 mm^58^.

### Site selection and fieldwork

Different terms have been used worldwide to describe peatlands with a certain ligneous vegetation cover (e.g. forested, treed, or wooded peatlands) as the terminology of these ecosystems is yet to be clarified. Here, the term “forested peatlands” refers to peatlands with trees over 4 m in height having a canopy coverage ≥ 25%^59^. The studied forested peatland site was selected based on previous studies conducted in the Clay Belt to ensure its regional representativeness^24,41,60^. Its selection was based on ecoforestry maps^61^ to identify black spruce–*Sphagnum*-dominated stands and field observations to choose a forested peatland presenting a range in organic layer thickness. The selected forested peatland (49°33′06′′N, 78°59′10′′O; Fig. 1b) has an organic layer thickness that varies between 40 cm and more than 1 m. The aboveground vegetation is largely dominated by black spruce and ericaceous shrubs, such as *Vaccinium angustifolium*, *Rhododendron groenlandicum*, *Kalmia angustifolia*, and *Chamaedaphne calyculata*. The understory is dominated by *Sphagnum* communities, particularly *S. angustifolium/fallax*.

Three sampling sites (CAS0, CAS50, CAS100) were established along a 100 m transect following a gradient of organic layer thickness (Fig. 2). Relative surface altitude and organic layer thickness were measured systematically at 5 m intervals along the transect using a high precision altimeter (ZIPLEVEL PRO-2000) and an Oakfield probe. Water table depths were measured at the same intervals a few hours after holes were dug to ensure that the water table level had stabilized. At each site, the diameter at breast height (DBH) of all trees (DBH ≥ 1 cm) within a 100 m^2^ plot (10 × 10 m) was measured.

One peat monolith was collected down to the mineral contact at the three sites using a Box corer^62^. Peat cores were retrieved from *Sphagnum*-dominated lawns that were representative of the mean peat thickness of the sites. Twenty black spruce trees were also selected at each site within a 10 m radius of the collected peat core. Peat thickness was measured at the bottom of each selected tree to make sure that it was representative of the mean peat thickness of the site. Only dominant trees with straight stem and no visible scars were selected. The DBH of selected trees was measured and cross-sections were collected at standard height (1.3 m).

### Peat core chronologies

Peat chronologies were developed from radiocarbon and ^210^Pb dating. A total of 11 samples were submitted to A. E. Lalonde AMS Laboratory (University of Ottawa, Canada) for accelerator mass spectrometry radiocarbon dating (^14^C). Peat initiation, the last fire event and main transitions in vegetation composition were dated for each core by carefully selecting plant macrofossil remains in the appropriate levels^26^. Results were calibrated with the IntCal13 calibration curve^63^. ^210^Pb dating was also performed on the uppermost 24–26 cm (above the fire horizon) of peat cores at 1 cm intervals by alpha spectrometry (EGG Ortec 476A) at the GEOTOP Research Center (Université du Québec à Montréal, Canada)^26^. Ages were inferred from the measurement of ^210^Po activity, using the constant rate of supply model^64^ following a HNO_3_-HCl-H_2_O_2_ digestion on samples^65^. Lead-210 dating has been very rarely conducted in forested peatlands but our results suggest that ^210^Pb measurements perform well in these ecosystems, as they agree with ^14^C dates. Age-depth models were generated with *rbacon* package in R (version 2.3.9.1)^66^. Ages are expressed in calendar years before present (cal yr BP; 1950 CE) and the peat surface age is therefore set to − 67 cal year BP (coring year: 2017 CE).

### Identification of past fire events

Past local fire events were identified by analysing macroscopic charcoal fragments (> 0.5 mm) at 1 cm intervals along the three peat cores^26,67^. Subsamples of 1 cm^3^ were gently boiled with 10% KOH and washed through a 0.5 mm mesh sieve. Charcoal fragments were then counted in a gridded Petri dish under a stereomicroscope (10–40 × magnification).

### Tree-ring analysis

Dried cross-sections of the 60 sampled trees were finely sanded (from 80 to 600 grit size) before measuring ring widths along two radii with CooRecorder software (version 8.1.1)^68^. This procedure allowed the development of tree-ring chronologies and the characterisation of black spruce growth at each site. Samples were visually cross-dated using PAST5 software (version 5.0.610)^69^ and skeleton plots were generated with the R package *dplR* (version 1.6.9)^70^. Ring-width series were converted into annual cumulative biomass and then yearly averaged using all trees from the same site to compare tree growth along the paludification gradient.

### Carbon data from organic layers and tree biomass

Dry bulk density was determined at each centimeter in the three peat cores after drying overnight a 1 cm^3^ subsample at 105 °C. Organic matter density was then measured using loss-on-ignition at 550 °C for 3 h^71^. Peat C stocks were estimated by multiplying the amount of organic matter in peat cores by an estimated 50% carbon content mass^72^. Recent C stocks (last ~ 200 years) were calculated from the horizons above the last charcoal layer of each site. Long-term apparent rates of carbon accumulation (LORCA, g C m^−2^ year^−1^) were obtained by dividing the total mass of carbon accumulated by the basal ^14^C age at the organic-mineral interface. Recent apparent rates of carbon accumulation (RERCA, g C m-^2^ year^−1^) were calculated for the periods 1900 CE-present and 1950 CE-present by dividing the carbon mass accumulated by the age inferred from age-depth models. RERCA values were compared between sites, but cannot be compared with LORCA values as surface peat has undergone less decomposition than older peat^27^. Variations in apparent carbon accumulation rates (CAR, g C m^−2^ year^−1^) were estimated by dividing the C density (g cm^−3^) of each continuous centimeter by the deposition time (yr cm^−1^) generated by the age-depth modelling.

Tree C stocks were calculated from the aboveground and belowground biomass of every tree counted in the 100 m^2^ plot of each site. All trees within the study plots were black spruce trees. Individual aboveground biomass was estimated with DBH measurements using allometric equations adapted to black spruce growth^73^. Wood, bark, foliage and branches are all included in these estimates. Root biomass was estimated from three different equations^74–76^, using the DBH measurements or the tree aboveground biomass estimates. The C content of black spruce trees was assumed to be 50% of tree biomass^77^.

## Supplementary information

Supplementary Information.

Supplementary Dataset.

## Data Availability

The datasets generated and analysed during the current study are available in Supplementary Information files.

## References

[1] Le Quéré C (2014). Global carbon budget 2014. Earth Syst. Sci. Data.

[2] Pan Y (2011). A large and persistent carbon sink in the world's forests. Science.

[3] Hugelius G (2020). Large stocks of peatland carbon and nitrogen are vulnerable to permafrost thaw. Proc. Natl. Acad. Sci. USA.

[4] Payette S, Payette S, Rochefort L (2001). Les principaux types de tourbières. Écologie des tourbières du Québec-Labrador.

[5] Charman D (2002). Peatlands and Environmental Change.

[6] Pakarinen P (1995). Classification of boreal mires in Finland and Scandinavia: a review. Vegetatio.

[7] Zoltai SC, Martikainen PJ, Apps MJ, Price DT (1996). Estimated extent of forested peatlands and their role in the global carbon cycle. Forest Ecosystems, Forest Management and the Global Carbon Cycle.

[8] Lavoie M, Paré D, Bergeron Y (2005). Impact of global change and forest management on carbon sequestration in northern forested peatlands. Environ. Rev..

[9] Thompson DK, Simpson BN, Beaudoin A (2016). Using forest structure to predict the distribution of treed boreal peatlands in Canada. For. Ecol. Manag..

[10] Webster K (2018). Spatially-integrated estimates of net ecosystem exchange and methane fuxes from Canadian peatlands. Carbon Balance Manag..

[11] Minkkinen, K., Byrne, K. A. & Trettin, C. Climate impacts of peatland forestry in *Peatlands and Climate Change* (ed. Strack, M.) 98–122 (International Peat Society, 2008).

[12] Laiho R, Laine J (1997). Tree stand biomass and carbon content in an age sequence of drained pine mires in southern Finland. For. Ecol. Manag..

[13] Minkkinen K, Laine J (1998). Long-term effect of forest drainage on the peat carbon stores of pine mires in Finland. Can. J. For. Res..

[14] Lohila A (2011). Greenhouse gas flux measurements in a forestry-drained peatland indicate a large carbon sink. Biogeosciences.

[15] Simola H, Pitkänen A, Turunen J (2012). Carbon loss in drained forestry peatlands in Finland, estimated by re-sampling peatlands surveyed in the 1980s. Eur. J. Soil Sci..

[16] Griscom BW (2017). Natural climate solutions. Proc. Natl. Acad. Sci. USA.

[17] Yosef G (2018). Large-scale semi-arid afforestation can enhance precipitation and carbon sequestration potential. Sci. Rep..

[18] Bastin J-F (2019). The global tree restoration potential. Science.

[19] Lewis SL, Wheeler CE, Mitchard ETA, Koch A (2019). Restoring natural forests is the best way to remove atmospheric carbon. Nature.

[20] IPCC. Climate Change and Land: an IPCC special report on climate change, desertification, land degradation, sustainable land management, food security, and greenhouse gas fluxes in terrestrial ecosystems (eds. Shukla, P.R., Skea, J., Calvo Buendia, E., Masson-Delmotte, V., Pörtner, H.-O., Roberts, D. C., Zhai, P., Slade, R., Connors, S., van Diemen, R., Ferrat, M., Haughey, E., Luz, S., Neogi, S., Pathak, M., Petzold, J., Portugal Pereira, J., Vyas, P., Huntley, E., Kissick, K., Belkacemi, M. & Malley, J.), https://www.ipcc.ch/srccl/ (IPCC, 2019).

[21] Bona KA, Fyles JW, Shaw C, Kurz WA (2013). Are mosses required to accurately predict upland black spruce forest soil carbon in national-scale forest C accounting models?. Ecosystems.

[22] Leifeld J, Menichetti L (2018). The underappreciated potential of peatlands in global climate change mitigation strategies. Nat. Commun..

[23] Taillardat P, Thompson BS, Garneau M, Trottier K, Friess DA (2020). Climate change mitigation potential of wetlands and the cost-effectiveness of their restoration. Interface Focus.

[24] Magnan, G., Garneau, M., Le Stum-Boivin, É., Grondin, P. & Bergeron, Y. Long-term carbon sequestration in boreal forested peatlands in eastern Canada. *Ecosystems* (2020).

[25] Laamrani A, DesRochers A, Blackburn L (2016). Effect of organic layer thickness on black spruce aging mistakes in Canadian boreal forests. Forests.

[26] Beaulne, J., Boucher, É., Garneau, M. & Magnan, G. Paludification reduces black spruce growth rate but does not alter tree water use efficiency in Canadian boreal forested peatlands. *For. Ecosyst.*10.21203/rs.3.rs-57461/v2. 10.1186/s40663-021-00307-x PMC8550502 34721933

[27] Young Y (2019). Misinterpreting carbon accumulation rates in records from near-surface peat. Sci Rep.

[28] Jacobs J, Work T, Paré D, Bergeron Y (2015). Paludification of boreal soils reduces wood decomposition rates and increases wood-based carbon storage. Ecosphere.

[29] Joosten H, Sirin A, Couwenberg J, Laine J, Smith P, Bonn A, Allott T, Evans M, Joosten H, Stoneman R (2016). The role of peatlands in climate regulation. Peatland Restoration and Ecosystem Services: Science, Policy and Practice.

[30] Shetler G, Turetsky MR, Kane E, Kasischke E (2008). *Sphagnum* mosses limit total carbon consumption during fire in Alaskan black spruce forests. Can. J. For. Res..

[31] Terrier A, de Groot WJ, Girardin MP, Bergeron Y (2014). Dynamics of moisture content in spruce-feather moss and spruce-*Sphagnum* organic layers during an extreme fire season and implications for future depths of burn in Clay Belt black spruce forests. Int. J. Wildland Fire.

[32] Moore T, Bubier J, Bledzki L (2007). Litter decomposition in temperate peatland ecosystems: the effect of substrate and site. Ecosystems.

[33] Lang SI (2009). An experimental comparison of chemical traits and litter decomposition rates in a diverse range of subarctic bryophyte, lichen and vascular plant species. J. Ecol..

[34] Manies KL, Harden JW, Fuller CC, Turetsky MR (2016). Decadal and long-term boreal soil carbon and nitrogen sequestration rates across a variety of ecosystems. Biogeosciences.

[35] Loisel J (2014). A database and synthesis of northern peatland soil properties and Holocene carbon and nitrogen accumulation. Holocene.

[36] Garneau M (2014). Holocene carbon dynamics of boreal and subarctic peatlands from Québec, Canada. Holocene.

[37] Magnan G (2019). Holocene vegetation dynamics and hydrological variability in forested peatlands of the Clay Belt, eastern Canada, reconstructed using a palaeoecological approach. Boreas.

[38] Turunen J, Roulet NT, Moore TR, Richard PJH (2004). Nitrogen deposition and increased carbon accumulation in ombrotrophic peatlands in eastern Canada. Glob. Biogeochem. Cycles.

[39] Piilo SR (2019). Recent peat and carbon accumulation following the Little Ice Age in northwestern Québec, Canada. Environ. Res. Lett..

[40] Heikurainen L (1964). Improvement of forest growth on poorly drained peat soils. Int. Rev. For. Res..

[41] Simard M, Lecomte N, Bergeron Y, Bernier PY, Paré D (2007). Forest productivity decline caused by successional paludification of boreal soils. Ecol. Appl..

[42] Pluchon N, Hugelius G, Kuusinen N, Kuhry P (2014). Recent paludification rates and effects on total ecosystem carbon storage in two boreal peatlands of Northeast European Russia. Holocene.

[43] Päivänen, J. The effects of silvicultural treatments on the ground water table in Norway spruce and Scots pine stands on peat in *Proceedings of the 6*^*th*^* International Peat Congress* (ed. International Peat Society) 433–438 (International Peat Society, 1980).

[44] Lappalainen, E. Peatlands and peat resources in Finland in *Peatlands in Finland* (ed. Vasander, H.) 36–38 (Finnish Peatland Society, 1996).

[45] Lavoie M, Paré D, Fenton N, Groot A, Taylor K (2005). Paludification and management of forested peatlands in Canada: a literature review. Environ. Rev..

[46] Lafleur B, Paré D, Fenton NJ, Bergeron Y (2011). Growth of planted black spruce seedlings following mechanical site preparation in boreal forested peatlands with variable organic layer thickness: 5-year results. Ann. For. Sci..

[47] Prévost M, Dumais D (2018). Long-term growth response of black spruce advance regeneration (layers), natural seedlings and planted seedlings to scarification: 25th year update. Scand. J. For. Res..

[48] Ojanen P, Minkkinen K, Penttilä T (2013). The current greenhouse gas impact of forestry-drained boreal peatlands. For. Ecol. Manag..

[49] Kurz WA (2013). Carbon in Canada’s boreal forest—A synthesis. Environ. Rev..

[50] Lafleur B (2018). Ecosystem management in paludified boreal forests: enhancing wood production, biodiversity, and carbon sequestration at the landscape level. For. Ecosyst..

[51] Peñuelas J, Canadell JG, Ogaya R (2011). Increased water-use efficiency during the 20th century did not translate into enhanced tree growth. Glob. Ecol. Biogeogr..

[52] Lévesque M, Siegwolf R, Saurer M, Eilmann B, Rigling A (2014). Increased water-use efficiency does not lead to enhanced tree growth under xeric and mesic conditions. New Phytol..

[53] van der Sleen P (2014). No growth stimulation of tropical trees by 150 years of CO_2_ fertilization but water-use efficiency increased. Nat. Geosci..

[54] Giguère-Croteau C (2019). North America’s oldest boreal trees are more efficient water users due to increased [CO2], but do not grow faster. Proc. Natl. Acad. Sci. USA.

[55] Saucier, J.-P., Robitaille, A. & Grondin, P. Cadre bioclimatique du Québec in *Manuel de foresterie,* 2nd ed. (eds. Doucet, R. & Côté, M.) 186–205 (Éditions MultiMondes, 2009).

[56] Vincent J-S, Hardy L (1977). L’évolution et l’extension des lacs glaciaires Barlow et Ojibway en territoire québécois. Géogr. Phys. Quat..

[57] Bergeron Y, Gauthier S, Flannigan MD, Kafka V (2004). Fire regimes at the transition between mixedwood and coniferous boreal forest in northwestern Quebec. Ecology.

[58] McKenney DW (2011). Customized spatial climate models for North America. Bull. Am. Meteorol. Soc..

[59] Bazoge, A., Lachance, D. & Villeneuve, C. Identification et délimitation des milieux humides du Québec méridional, Ministère du Développement durable, de l’Environnement et de la Lutte contre les changements climatiques, Direction de l’écologie et de la conservation et Direction des politiques de l’eau (Gouvernement du Québec, 2014).

[60] Le Stum-Boivin É (2019). Spatiotemporal evolution of paludification associated with autogenic and allogenic factors in the black spruce–moss boreal forest of Québec, Canada. Quat. Res..

[61] MFFP (Ministère des Forêts, de la Faune et des Parcs). Ecoforestry maps, ftp://transfert.mffp.gouv.qc.ca/Public/Diffusion/DonneeGratuite/Foret/DONNEES_FOR_ECO_SUD/Resultats_inventaire_et_carte_ecofor (2019).

[62] Jeglum, J. K., Rothwell, R. L., Berry, G. J. & Smith, G. K. M. A Peat Sampler for Rapid Survey. Frontline, Technical Note 13, 921–932 (Canadian Forestry Service, 1992).

[63] Reimer PJ (2013). IntCal13 and MARINE13 radiocarbon age calibration curves 0–50000 years calBP. Radiocarbon.

[64] Appleby PG, Oldfield F (1978). The calculation of ^210^Pb dates assuming a constant rate of supply of unsupported ^210^Pb to the sediment. CATENA.

[65] Ali AA, Ghaleb B, Garneau M, Asnong H, Loisel J (2008). Recent peat accumulation rates in minerotrophic peatlands of the Bay James region, Eastern Canada, inferred by ^210^Pb and ^137^Cs radiometric techniques. Appl. Radiat. Isot..

[66] Blaauw, M. & Christen, J. A. rbacon: Age-Depth Modelling using Bayesian Statistics. R package version 2.3.9.1, https://CRAN.R-project.org/package=rbacon (2019).

[67] Magnan G, Lavoie M, Payette S (2012). Impact of fire on long-term vegetation dynamics of ombrotrophic peatlands in northwestern Québec, Canada. Quat. Res..

[68] Larson, L.-A. CooRecorder: image co-ordinate recording, version 8.1.1, http://www.cybis.se (Cybis, 2016).

[69] Knibbe, B. PAST5: Personal Analysis System for Treering Research, version 5.0.610, http://www.sciem.com/products/past/ (SCIEM, 2019).

[70] Bunn, A. *et al*. dplR: Dendrochronology Program Library in R. R package version 1.6.9, https://CRAN.R-project.org/package=dplR (2018).

[71] Dean EW (1974). Determination of carbonate and organic matter in calcareous sediments and sedimentary rocks by loss on ignition: comparison with other methods. J. Sediment. Petrol..

[72] Chambers FM, Beilman DW, Yu Z (2011). Methods for determining peat humification and for quantifying peat bulk density, organic matter and carbon content for palaeostudies of climate and peatland carbon dynamics. Mires Peat.

[73] Ung CH, Bernier P, Guo XJ (2008). Canadian national biomass equations: new parameter estimates that include British Columbia data. Can. J. For. Res..

[74] Kurz WA, Beukema SJ, Apps MJ (1996). Estimation of root biomass and dynamics for the carbon budget model of the Canadian forest sector. Can. J. For. Res..

[75] Ouimet R, Camiré C, Brazeau M, Moore J-D (2008). Estimation of coarse root biomass and nutrient content for sugar maple, jack pine, and black spruce using stem diameter at breast height. Can. J. For. Res..

[76] Brassard BW, Chen HYH, Bergeron Y, Paré D (2011). Coarse root biomass allometric equations for *Abies balsamea*, *Picea mariana*, *Pinus banksiana*, and *Populus tremuloides* in the boreal forest of Ontario, Canada. Biomass Bioenergy.

[77] Thomas SC, Martin AR (2012). Carbon content of tree tissues: a synthesis. Forests.

