# Supplementary material for: Peat deposits store more carbon than trees in forested peatlands of the boreal biome
Source: Sci Rep. 2021 Jan 29;11:2657. doi: 10.1038/s41598-021-82004-x (PMC7846601; doi:10.1038/s41598-021-82004-x)
Supplement: Supplementary file 1 — Supplementary Information. [file 41598_2021_82004_MOESM1_ESM.pdf]

Peat deposits store more carbon than trees in forested peatlands of the boreal biome

Joannie Beaulne[1,2,3,*], Michelle Garneau[1,2,3,4,*], Gabriel Magnan[1,3], and Étienne Boucher[1,2,4]

[1] Geotop Research Center, Université du Québec à Montréal, Montréal, Québec H3C 3P8, Canada

[2] Department of Geography, Université du Québec à Montréal, Montréal, Québec H3C 3P8, Canada

[3] GRIL-UQAM, Université du Québec à Montréal, Montréal, Québec H3C 3P8, Canada

[4] Centre d'études nordiques, Université Laval, Québec, Québec G1V 0A6, Canada

* Emails: joannie.beaulne@gmail.com; garneau.michelle@uqam.ca

Supplementary Information

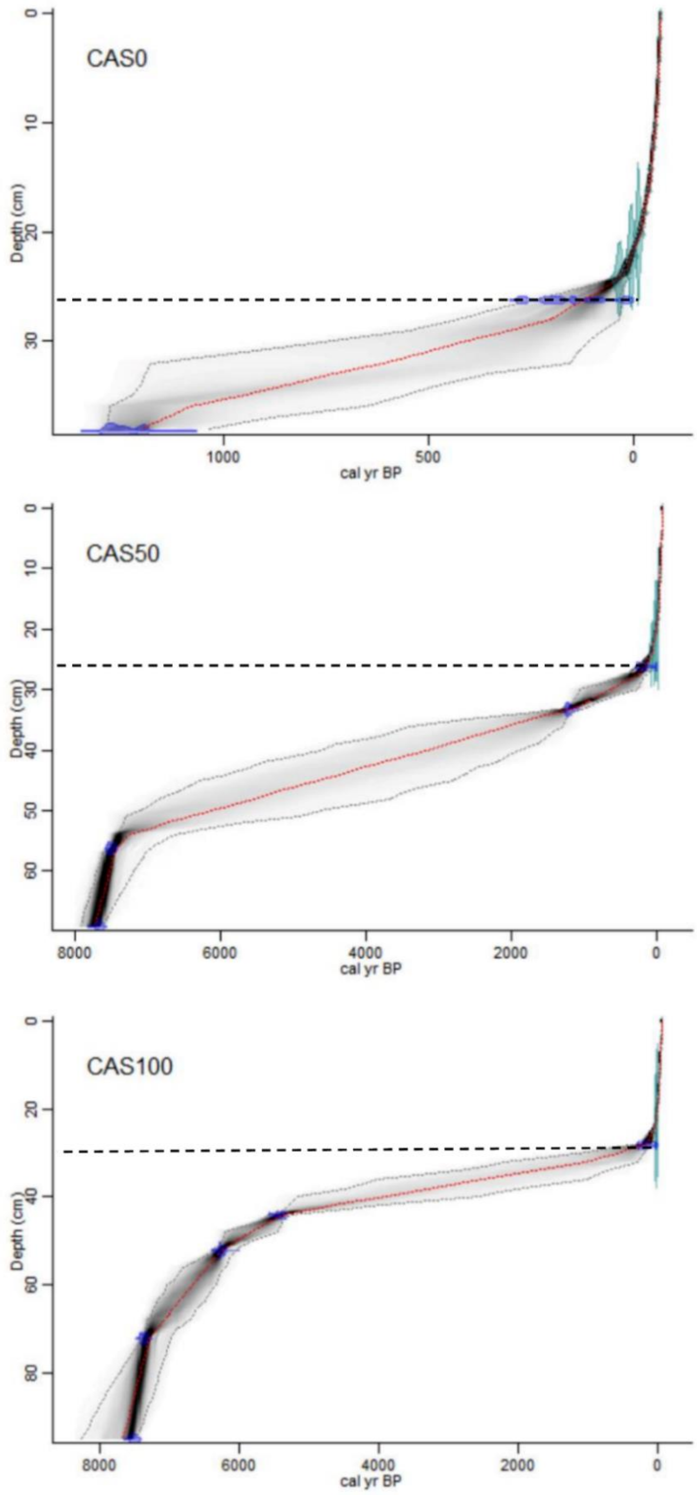

Figure S1. Age-depth models for each peat core developed using the *rbacon* package in R (version 2.3.9.1) [66]. Dotted grey lines indicate the 95% confidence intervals and the blue shapes show the calibrated ages. Dashed lines indicate the last fire event.

Table S1. Radiocarbon (^{14}C) date results [26].

Core	Depth (cm)	Laboratory number	Material dated	Radiocarbon age±1σ (^{14}C yr BP)	Calibrated age range (cal yr BP)	Median calibrated age (cal yr BP)
CAS0	26-27	UOC-9614	Charcoal, charred needles	158±29	0-285	175
	38-39	UOC-8508	Bulk peat	1314±32	1182-1296	1255
CAS50	26-27	UOC-9615	Charcoal, charred needles	171±29	0-290	179
	33-34	UOC-8509	Charcoal, charred needles	1264±24	1175-1281	1224
	56-57	UOC-9617	*Sphagnum* stems	6610±29	7440-7565	7502
	69-70	UOC-9616	Bulk peat	6838±29	7610-7724	7667
CAS100	28-29	UOC-8512	Charcoal, charred needles	154±24	35-284	178
	44-45	UOC-8513	*Picea* needles, *Sphagnum* stems	4691±31	5320-5577	5397
	52-53	UOC-8514	*Picea* needles, *Sphagnum* stems	5469±38	6190-6386	6274
	72-73	UOC-8515	*Picea* and *Larix* needles	6466±34	7311-7436	7374
	95-96	UOC-6053	Bulk peat	6635±41	7441-7578	7522

Table S2. Results of ^{210}Pb dating [26].

Core	Depth (cm)	^{210}Pb activity (Bq/kg)	±	Year (CE)
CAS0	0.5	79.41	4.18	2017
	2.5	102.46	5.06	2016
	4.5	98.37	4.59	2014
	6.5	108.74	5.31	2012
	8.5	157.25	6.27	2010
	10.5	168.81	6.80	2005
	12.5	137.72	7.78	2000
	14.5	130.63	6.12	1994
	16.5	114.32	6.35	1987
	18.5	130.76	5.92	1978
	20.5	103.92	4.71	1964
	22.5	72.40	3.21	1946
	24.5	65.74	3.11	1918
CAS50	0.5	186.87	7.92	2016
	2.5	275.90	15.64	2014
	4.5	307.80	16.67	2010
	6.5	354.92	14.12	2005
	8.5	236.38	8.79	1999
	10.5	264.91	10.47	1992
	12.5	202.29	8.78	1985
	14.5	152.45	7.83	1978
	16.5	198.63	9.85	1970
	18.5	198.52	9.65	1957
	20.5	148.80	6.53	1930
	22.5	67.59	3.01	1898
	24.5	46.27	2.30	1863
CAS100	0.5	225.04	8.27	2016
	2.5	220.32	8.00	2013
	4.5	226.30	7.99	2009
	6.5	221.53	7.84	2005
	8.5	176.87	6.46	1999
	10.5	171.00	6.56	1993
	12.5	119.42	5.11	1987
	14.5	129.84	5.45	1981
	16.5	142.69	5.48	1975
	18.5	132.21	4.88	1966
	20.5	99.21	4.18	1952
	22.5	65.17	2.74	1935
	24.5	53.84	2.34	1918
	26.5	26.61	1.48	1891
